# Prediction of tumor-specific splicing from somatic mutations as a source of neoantigen candidates

**DOI:** 10.1093/bioadv/vbae080

**Published:** 2024-05-29

**Authors:** Franziska Lang, Patrick Sorn, Martin Suchan, Alina Henrich, Christian Albrecht, Nina Köhl, Aline Beicht, Pablo Riesgo-Ferreiro, Christoph Holtsträter, Barbara Schrörs, David Weber, Martin Löwer, Ugur Sahin, Jonas Ibn-Salem

**Affiliations:** TRON—Translational Oncology at the University Medical Center of Johannes Gutenberg University Mainz gGmbH, Mainz 55131, Germany; Faculty of Biology, Johannes Gutenberg University Mainz, Mainz 55128, Germany; TRON—Translational Oncology at the University Medical Center of Johannes Gutenberg University Mainz gGmbH, Mainz 55131, Germany; TRON—Translational Oncology at the University Medical Center of Johannes Gutenberg University Mainz gGmbH, Mainz 55131, Germany; TRON—Translational Oncology at the University Medical Center of Johannes Gutenberg University Mainz gGmbH, Mainz 55131, Germany; TRON—Translational Oncology at the University Medical Center of Johannes Gutenberg University Mainz gGmbH, Mainz 55131, Germany; TRON—Translational Oncology at the University Medical Center of Johannes Gutenberg University Mainz gGmbH, Mainz 55131, Germany; TRON—Translational Oncology at the University Medical Center of Johannes Gutenberg University Mainz gGmbH, Mainz 55131, Germany; TRON—Translational Oncology at the University Medical Center of Johannes Gutenberg University Mainz gGmbH, Mainz 55131, Germany; TRON—Translational Oncology at the University Medical Center of Johannes Gutenberg University Mainz gGmbH, Mainz 55131, Germany; TRON—Translational Oncology at the University Medical Center of Johannes Gutenberg University Mainz gGmbH, Mainz 55131, Germany; TRON—Translational Oncology at the University Medical Center of Johannes Gutenberg University Mainz gGmbH, Mainz 55131, Germany; TRON—Translational Oncology at the University Medical Center of Johannes Gutenberg University Mainz gGmbH, Mainz 55131, Germany; TRON—Translational Oncology at the University Medical Center of Johannes Gutenberg University Mainz gGmbH, Mainz 55131, Germany; BioNTech SE, Mainz 55131, Germany; Institute of Immunology, University Medical Center of the Johannes-Gutenberg University, Mainz 55131, Germany; TRON—Translational Oncology at the University Medical Center of Johannes Gutenberg University Mainz gGmbH, Mainz 55131, Germany

## Abstract

**Motivation:**

Neoantigens are promising targets for cancer immunotherapies and might arise from alternative splicing. However, detecting tumor-specific splicing is challenging because many non-canonical splice junctions identified in tumors also appear in healthy tissues. To increase tumor-specificity, we focused on splicing caused by somatic mutations as a source for neoantigen candidates in individual patients.

**Results:**

We developed the tool splice2neo with multiple functionalities to integrate predicted splice effects from somatic mutations with splice junctions detected in tumor RNA-seq and to annotate the resulting transcript and peptide sequences. Additionally, we provide the tool EasyQuant for targeted RNA-seq read mapping to candidate splice junctions. Using a stringent detection rule, we predicted 1.7 splice junctions per patient as splice targets with a false discovery rate below 5% in a melanoma cohort. We confirmed tumor-specificity using independent, healthy tissue samples. Furthermore, using tumor-derived RNA, we confirmed individual exon-skipping events experimentally. Most target splice junctions encoded neoepitope candidates with predicted major histocompatibility complex (MHC)-I or MHC-II binding. Compared to neoepitope candidates from non-synonymous point mutations, the splicing-derived MHC-I neoepitope candidates had lower self-similarity to corresponding wild-type peptides. In conclusion, we demonstrate that identifying mutation-derived, tumor-specific splice junctions can lead to additional neoantigen candidates to expand the target repertoire for cancer immunotherapies.

**Availability and implementation:**

The R package splice2neo and the python package EasyQuant are available at https://github.com/TRON-Bioinformatics/splice2neo and https://github.com/TRON-Bioinformatics/easyquant, respectively.

## 1 Introduction

Neoantigens are tumor-specific mutated gene products that are presented in form of neoepitopes on major histocompatibility complex (MHC) molecules and that are recognized by CD4^+^ or CD8^+^ T cells (Lang *et al.* 2022). Individualized cancer vaccines mediate successful anti-tumor responses by targeting these neoantigens (Sahin *et al.* 2017, Ott *et al.* 2017, Hilf *et al.* 2019, Keskin *et al.* 2019). Previous studies mainly focused on targeting neoantigens derived from single nucleotide variants (SNVs) as the most abundant mutation type. However, neoantigens can also derive from other mutation types such as short insertions and deletions (INDELs) or gene fusions which broaden the targetable neoantigen repertoire (Smith *et al.* 2019, Weber *et al.* 2022).

During mRNA splicing, multiple isoforms per gene may be created by removal of introns and joining of exons, increasing the functional diversity of the proteome (Rogalska *et al.* 2023). Importantly, 60% of the alternative splicing events are variable between tissues (Wang *et al.* 2008), and inter-individual variability contributes to individual phenotypes (Melé *et al.* 2015). The highly diverse nature of splicing was recently demonstrated by long-read sequencing of more than 70 000 novel transcripts in healthy tissues (Glinos *et al.* 2022).

Alternative splicing is specifically dysregulated in many tumors, impacting protein function and contributing to tumor heterogeneity (Climente-González *et al.* 2017, Kahles *et al.* 2018, Escobar-Hoyos *et al.* 2019, Öther-Gee Pohl and Myant 2022). For instance, disrupting mutations or expression changes of genes encoding RNA splicing regulators such as SF3B1 alter splicing specifically in tumors (Kahles *et al.* 2018, Escobar-Hoyos *et al.* 2019, Bigot *et al.* 2021, Oka *et al.* 2021, Öther-Gee Pohl and Myant 2022). Previous studies have shown that indeed novel peptides derived by alternative splicing in tumors can be presented by MHC-I and elicit cytotoxic CD8^+^ T-cell responses in human leukocyte antigen (HLA)-A24 transgenic mice (Oka *et al.* 2021), uveal melanoma (Bigot *et al.* 2021), acute myeloid leukemia (Ehx *et al.* 2021) and lung cancer (Merlotti *et al.* 2023). Alternative splicing can result in frameshifts of the translational reading frame, leading to mutated peptide sequences highly dissimilar to the wild-type proteome. Such dissimilarity could increase the likelihood of T-cell recognition and make them excellent targets.

However, it is challenging to ensure the tumor-specificity of such splicing events. A common strategy is to focus on splice junctions that are absent or low expressed in a set of matched or unmatched healthy tissues (Zhang *et al.* 2020, Ehx *et al.* 2021, Chai *et al.* 2022, Pan *et al.* 2023). However, given the great diversity and stochasticity of alternative splicing across healthy tissue, more evidence is required to consider splice junctions as truly tumor-specific candidates for individualized cancer vaccines (David *et al.* 2020).

Somatic mutations can directly alter splicing by disrupting canonical splicing motifs or creating novel splicing motifs (Jung *et al.* 2015, Jayasinghe *et al.* 2018, PCAWG Transcriptome Core Group et al. 2020, Cotto *et al.* 2023). Notably, integrating somatic mutations with splicing events in whole-genome sequenced pan-cancer cohorts suggested that 34% of the intronic mutations near exon–intron boundaries may affect splicing (PCAWG Transcriptome Core Group et al. 2020). Multiple computational tools predicting the effect of mutations on splicing and recent deep learning-based methods considerably improved splicing effect prediction (Wai *et al.* 2020, Riepe *et al.* 2021).

In contrast to splice junctions detected from RNA-seq alone, expressed splice junctions that are caused by somatic mutations can be considered as truly tumor-specific splice junctions. However, it is currently unclear whether such tumor-specific splice junctions can be predicted reliably from somatic mutations for individual cancer patients and how many splicing-derived neoantigen candidates qualify as promising targets for individualized cancer vaccines. Although several computational tools and pipelines exist to predict neoantigen candidates from alternative splicing (Chai *et al.* 2022, Pan *et al.* 2023), it remains challenging to use them with sufficient flexibility in custom workflows to analyze mutation effects on the level of splice junctions, integrate them with RNA-seq reads, and correct annotation on transcript and peptide levels.

In this study, we present two novel computational tools, splice2neo and EasyQuant, to facilitate the integration of the predicted effect of somatic mutations on splicing with splicing events detected from RNA-seq. By combining these tools in a novel analysis strategy, we identified a small but relevant set of tumor-specific splice junctions that can be interesting targets for individualized cancer vaccines.

## 2 Methods

### 2.1 Whole exome sequencing and somatic mutation calling

Whole exome sequencing (WES) and somatic mutation calling is described in the Supplementary methods.

### 2.2 Predicting the effect of somatic mutations on splicing

The effect of somatic mutations on splicing was predicted with SpliceAI (Jaganathan *et al.* 2019) (v1.3.1) and MMSplice (Cheng *et al.* 2019) (v2.1.1). For SpliceAI, the GENCODE V24 canonical annotation (grch37) included in the package was used. GENCODE v34lift37 was used as annotation for MMSplice. Both tools were run with default settings.

### 2.3 Identifying alternative splicing events from RNA-seq

RNA-seq reads were aligned to the hg19 reference genome with STAR (Dobin *et al.* 2012) (v2.7.0a). The options “twopassMode Basic—outSAMstrandField intronMotif” were used to generate BAM files for the tool LeafCutter based on recommendation in the manual. Alternative splicing events were detected with SplAdder (Kahles *et al.* 2016) (v3.0.2) and LeafCutter (Li *et al.* 2018) (v0.2.9). Splicing events from exon skipping (ES), alternative 3’ or 5’ splice sites (A3SS, A5SS), intron retention (IR) and mutually exclusive exons were identified with SplAdder, ignoring mismatch information in the alignment file and using GENCODE (v34lift37) annotation. Splicing events were identified with LeafCutter annotation-free using the settings as recommended in the manual (-m 50 -l 500000—checkchrom). Here, the tool how_are_we_stranded_here (Signal and Kahlke 2022) (v1.0.1) was used to computationally determine the strandedness of the RNA-seq and regtools (Cotto *et al.* 2023) (v0.5.2) was used to extract junctions using the recommended parameters of -a 8 -m 50 -M 500000. The union of RNA-seq derived junctions was considered per sample if RNA-seq replicates were available.

### 2.4 Splice2neo: prediction of splice junctions and derived mutated transcript and peptide sequences

We developed the R-package splice2neo (https://github.com/TRON-Bioinformatics/splice2neo) to identify mutation-retrieved splice junctions with RNA-seq support.

Splice2neo can identify splice junctions defined by the chromosome (chr), the last genomic position of the left exon (start), the first base of the right exon (end) and the transcriptional direction (strand) in the format “chr: start-end: strand” directly from the output of multiple tools. The R-package relies mainly on the functionalities of the Bioconductor packages GenomicRanges (Lawrence *et al.* 2013) and Biostrings (Pagès et al.). Splice2neo converts the raw output from LeafCutter, SplAdder, MMSplice, and SpliceAI into resulting splice junctions.

LeafCutter and SplAdder provide the genomic coordinates of the identified splicing events. Splice2neo transforms all events into the standardized junction format covering all potential splice junctions including canonical junctions.

SpliceAI predicts the probability of a mutation on splicing and provides the position of the alternative splicing events relative to the mutation. Table 1 describes the rules on how splice2neo creates splice junctions based on the predicted effect by SpliceAI. Mutations can be covered by more than one gene and the splice effect of a somatic mutation may differ between genes depending of the position of the somatic mutation within the respective gene. Splice2neo optionally considers only transcripts related to the gene annotated by SpliceAI while predicting the effect on splicing.

**Table 1.** Rules on how splice junctions are created based on the mutation effect predicted by SpliceAI.

Change	Class	Left junction coordinate	Right junction coordinate
Donor loss	IR	pos	pos + strand_offset
Donor loss	ES	Upstream_end	downstream_start
Donor gain	A5SS	pos	downstream_start
Acceptor loss	IR	pos—strand_offset	pos
Acceptor loss	ES	Upstream_end	downstream_start
Acceptor gain	A3SS	Upstream_end	pos

MMSplice predicts the change on percent spliced in (PSI) for a given annotated exon. Splice2neo considers events with PSI score ≤ 0 as ES events and uses the delta_logit_psi output as mutation effect score. Here, splice junctions are built by the end of the upstream exon and the start of the downstream exon.

The table describes the rules on how the left and right coordinate of the splice junction (“chr: start-end: strand”) are determined based on the predicted effect from SpliceAI. Pos refers to the position that is predicted to be affected by the mutation by SpliceAI. Upstream_end and downstream_start refer to exon end or start coordinates. Splice junctions that relate to an intron retention event are defined by the rule “chr: pos-(pos + 1):strand” and must cover an exon-intron boundary. The strand_offset is +1 for transcript on the positive strand and −1 otherwise. IR: intron retention, ES: exon skipping, A5SS/A3SS: alternative 5’ or 3’ splice site.

Splice2neo provides function to annotate splice junctions with the resulting modified transcript and peptide sequences.

The transcript context sequence covers by default 200-bp exonic sequence up- and downstream of a splice junction from an alternative 5’ or 3’ splice site (A3SS, A5SS) or ES event (“cts_seq”). In case of IR events, the sequences cover the complete intronic sequence, flanked by 200-bp (default) exonic sequence up-and downstream of the IR event. The position of the splice junction in the sequence or the intron interval are given by “cts_junc_pos.” A unique id (“cts_id”) is given based on the context sequence and position as a XXH128 hash value.

Given the splice junction and coordinates of coding sequences (CDS) of reference transcripts, splice2neo computes the resulting protein sequence for each affected transcript and the relevant peptide context sequence. First, wild-type CDSs are modified according to the junction coordinates and translated into protein sequences. A frameshift is annotated, if the wild-type and the modified CDS have a length difference that is not a multiple of three. Given the junction position in the modified CDS, we determine whether an event follows the first or the second/third reading frame. Then, we used the modified and wild-type protein sequence, the junction positions in the CDS and protein, and reading frame and frameshift information to extract the relevant peptide context sequences from the modified protein sequence. This relies on the calculation of a “normalized” position of the junction in the protein which is defined as the amino acid of the last wild-type amino acid in the mutated on the left side of the event. In cases of in-frame events, this peptide context sequence covers junctions with potentially novel amino acid sequences (residue at the junction position or inserted novel residues) flanked with 13 wild-type amino acids up- and downstream. Frameshift peptides are flanked by an upstream wild-type region and are translated until the next stop codon. If splice junctions do not generate mutated gene products, no peptide context sequence is provided. This can be the case if junction does not affect the CDS, or the “mutated” gene product is only a truncated version of the wild-type CDS.

In this study, we used splice2neo to convert the raw output from LeafCutter, SplAdder, MMSplice, and SpliceAI into resulting splice junctions. While converting the output of SpliceAI with splice2neo, only transcripts related to the annotated gene were considered. Using splice2neo, we further annotated splice junctions with the resulting modified transcript and peptide sequences. Furthermore, splice junctions were annotated whether they are canonical using a database of canonical splice junctions built from GENCODE v34lift37 using the R-package GenomicFeatures (Lawrence *et al.* 2013) (v.1.46.5) and classified as “normal” if they were contained in a dataset of normal splice junctions previously identified by Jaganathan *et al.* (2019) with LeafCutter in 1740 RNA-seq samples of 53 healthy tissues from Gene and Tissue Expression (GTEx). We used splice2neo v0.6.2 in this study.

### 2.5 Requantification of RNA-seq reads for mutation-retrieved splice junctions

To quantify the number of supporting RNA-seq reads for given splice junctions in a sensitive and targeted manner, we developed and applied EasyQuant (v0.4.0) https://github.com/TRON-Bioinformatics/easyquant. Analogous to the requantification for gene fusions that we previously developed in EasyFuse (Weber *et al.* 2022), EasyQuant aligns reads to a context sequence (“cts_seq”) constructed from the splice junction and calculates the reads supporting the junction (Supplementary Fig. S1). RNA-seq reads are mapped to the context sequence with STAR (Dobin *et al.* 2012) with the following parameters:—outFilterMismatchNoverReadLmax 0.015—alignEndsType EndToEnd—outFilterMultimapNmax -1—outSAMattributes NH HI AS nM NM MD—scoreDelBase -2—scoreInsBase -2. The STAR parameter scoreDelOpen, scoreInsOpen and outFilterMismatchNoverReadLmax can be defined by the user in the configuration file of EasyQuant by providing indel_open_penalty, indel_extension_penalty, and mismatch_ratio, respectively in the section “general.”

Next, the context sequence is divided into intervals based on the provided positions. For each interval, the number of reads within the interval is counted as well as reads overlapping interval end positions. Junctions from alternative splice sites or exon-skipping events are divided into two intervals. The end of the first interval represents the splice junction of interest. Reads covering the junction position by at least 10 bp are considered to support the splice junction (“junction reads”). Context sequences for IRs are divided into three intervals, whereby the second interval is the retained intron. We summarized the coverage of the entire intron by the median number of reads per position in the interval. The output values from EasyQuant are defined in Table 2.

**Table 2.** Read quantification in EasyQuant per interval and input sequence.

Value	Definition
overlap_interval_end_reads	Number of reads that overlap interval end position by at least 10 bp
span_interval_end_pairs	Number of read pair that span the interval end. Each read of the pair must be map on a different interval.
within_interval	Number of read that maps completely within the interval.
coverage_perc	Percent of interval positions that are covered by at least one read read.
coverage_mean	Mean number of mapped reads per interval position.

If RNA-seq replicates were available, read counts and summary values were summed up per splice junction. To not overestimate IR read support, we considered here only potential IR events for which the retained intron region did not overlap with any exon of any other transcript.

EasyQuant was run in the interval mode (—interval_mode) and reads covering the junction position by at least 10 bp were considered to support the splice junctions (-d 10). RNA-seq reads were mapped to the context sequence with STAR (Dobin *et al.* 2012) (-m star). The following STAR parameters were provided in the config file of Easyquant in the general section mismatch_ratio = 0.015 (outFilterMismatchNoverReadLmax), indel_open_penalty = −1000 (scoreDelOpen), indel_extension_penalty = −2 (scoreInsOpen).

### 2.6 False discovery rate estimation for prediction of target splice junctions

We aimed to derive a detection rule to predict splice junctions derived from somatic mutations as tumor-specific targets in a discovery cohort of 85 melanoma patients (Van Allen *et al.* 2015, Riaz *et al.* 2017) and to confirm it in a verification cohort of 27 melanoma patients (Hugo *et al.* 2016).

To identify potential false positive splice junctions, we compared the RNA-seq support of novel mutation-retrieved splice junctions from the actual sample with the RNA-seq support from a different individual who does not has the corresponding mutation. The permutation analysis was performed among all samples per study [e.g. within Van Allen *et al.* (2015) and within Riaz *et al.* (2017) separately]. Predicted splice junctions from somatic mutations were requantified with EasyQuant in the RNA-seq of all other independent tumor samples. Second, mutation-retrieved splice junctions were searched among the RNA-seq derived junctions identified in other biological independent samples.

A mutation-retrieved splice junction with RNA-seq support was labelled as false positive if there was support by requantification or SplAdder/LeafCutter derived splice junctions in at least one other independent sample. The estimated false discovery rate (FDR) was defined as the number of such false positives divided by all considered candidate splice junctions in the discovery or verification cohort.

To identify an appropriate detection rule, candidate splice junctions were gradually filtered by a grid of thresholds on the mutation effect scores from MMSplice and SpliceAI [0, 0.05, 0.1, 0.15, 0.2, 0.25, 0.3, 0.35, 0.4, 0.5, 0.6, 0.7, 0.8, 0.9], and the requantification read support [1, 2, 3, 4]. The number of remaining splice junctions and the FDR was estimated for each threshold combination. The threshold combination with the lowest FDR in the discovery cohort was defined as the detection rule and remaining candidate splice junctions were defined as targets. This detection rule was applied to the verification cohort to estimate the resulting number of target splice junctions and the final estimated FDR.

### 2.7 Annotation of neoantigen candidates

MHC-I and -II genotypes were determined with HLA-HD (Kawaguchi *et al.* 2017) (v1.2.0.1) for each patient based on the WES-seq data of the normal sample. NeoFox (Lang *et al.* 2021) (v1.0.2) was used to annotate neoantigen candidates derived by target splice junctions with neoantigen features. Here, the predicted mutated peptide sequences without information about variant allele frequency or transcript expression was provided as input to NeoFox. Furthermore, MHC-I and -II genotypes of the patients were provided to the tool as input.

### 2.8 Confirmation of splice junction expression with qRT-PCR

Primer design and confirmation of splice junctions with qRT-PCR is described in Supplementary Methods.

## 3 Results

### 3.1 Splice2neo allows diverse analysis of splice junctions from mutation effects and RNA-seq

**Figure 1.**
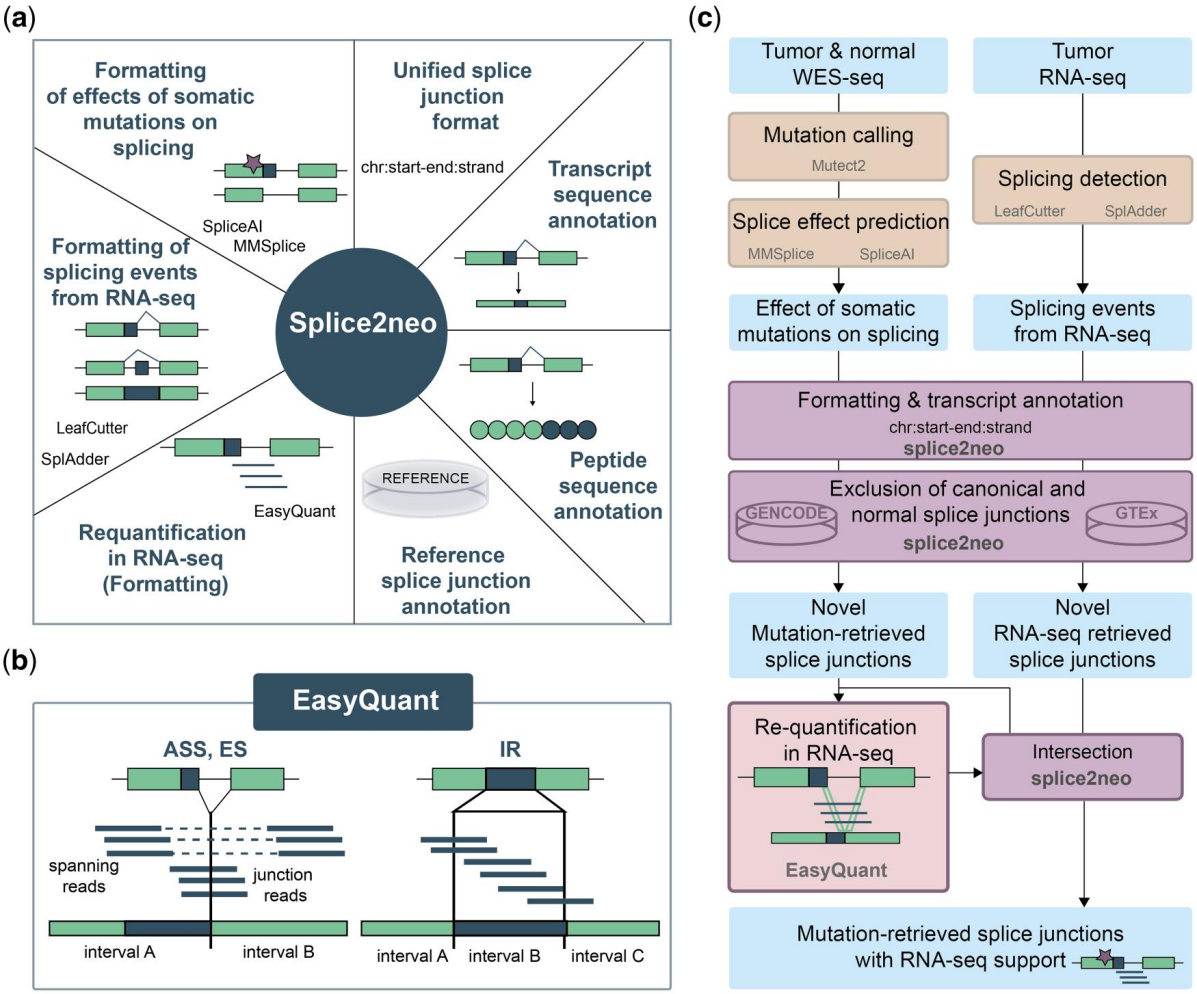
The tools splice2neo and EasyQuant provide diverse functions for identifying and analyzing of splice junctions as a source of neoantigen candidates. (a) Overview of functionalities implemented in the splice2neo R package. Splice2neo formats the output of several splicing tools into a unified junction format “chr: start-end: strand,” can exclude canonical or normal splice junctions (e.g. from GENCODE or GTEx) and annotates altered transcript and peptide sequences. (b) EasyQuant implements a targeted mapping approach to quantify RNA-seq reads that support a splice junction or retained intron. (c) Workflow to detect candidate splice junctions. The effect of somatic mutations on splicing was predicted with SpliceAI and MMSplice, and expressed splicing events were detected with LeafCutter and SplAdder for a given tumor sample, followed by formatting with splice2neo into the unified splice junction format. Novel mutation and RNA-seq retrieved splice junctions were intersected to identify mutation-retrieved splice junctions with RNA-seq support as candidates. To expand the number of candidates, novel mutation-derived were requantified in tumor RNA-seq with EasyQuant in a targeted manner.

First, we developed the R package splice2neo as a software library with multiple functions to integrate the effect of somatic mutations on splicing with splicing events detected in RNA-seq for the prediction of neoantigen candidates (Fig. 1a). Splice2neo leverages functionalities from Bioconductor packages, such as GenomicRanges (Lawrence *et al.* 2013), to model genomic locations and associated annotations. Splice2neo provides several functions to parse and format the output of multiple mutation effect prediction tools [MMSplice (Cheng *et al.* 2019), SpliceAI (Jaganathan *et al.* 2019)] and splicing detection tools [SplAdder (Kahles *et al.* 2016), LeafCutter (Li *et al.* 2018)] in a unified splice junction format, defined by the genomic coordinates of the resulting splice junction positions. For example, the mutation effect tool SpliceAI outputs a VCF file with loss and gain predictions of splicing donors and acceptors. These mutation effects can be parsed with the function “parse_spliceai()” and combined with reference transcripts in the function “annotate_mut_effect()” to compute the resulting splicing event types for each potentially affected transcript and resulting splice junctions. The splice event types include A3SS, A5SS, ES, and IRs. Further functions for splice junction analysis include the annotation of given splice junctions with putatively affected transcripts [“add_tx()”], the resulting transcript sequences [“add_context_seq()”], as well as, the resulting peptide sequences [“add_peptide()”]. The R-package is designed as a modular and easy-to-expand library and contains a range of independent functionalities for customized splice junction analysis on a joined dataset of multiple samples or for individual samples (Fig. 1a).

### 3.2 EasyQuant implements targeted requantification of splice junctions in RNA-seq

The unbiased *de novo* identification of splice junctions from short-read RNA-seq is a challenging task and benchmarking studies of splicing detection tools showed low overlap between tools (Mehmood *et al.* 2020, Jiang *et al.* 2023). We reasoned that targeted mapping of RNA-seq reads to the candidate splice junction sequence, as reference, allows more accurate and sensitive assessment of the RNA-seq read support per junction. Previously, we demonstrated accurate requantification of gene fusion candidates (Weber *et al.* 2022). In this study, we implemented EasyQuant, a dedicated tool for targeted requantification of reads covering a presumed splice junction or a retained intron of interest (Fig. 1b). EasyQuant is written in python and expects as input the transcript context sequences around a splice junction together with the interval-defining coordinates which can be constructed by splice2neo. Context sequences of ES and alternative splice sites are divided into two intervals flanking the junction position. Context sequences of retained introns are divided into three intervals of which the middle interval represents the retained intron. RNA-seq reads from fastq or bam files are aligned with stringent settings to the context sequence. EasyQuant returns a table with the number of mapped reads and read pairs that overlap interval ends and interval ranges and thereby support the splice junction or retained intron in question (Supplementary Fig. S1).

### 3.3 Identification of mutation-retrieved splice junctions with support in matched tumor RNA-seq

**Figure 2.**
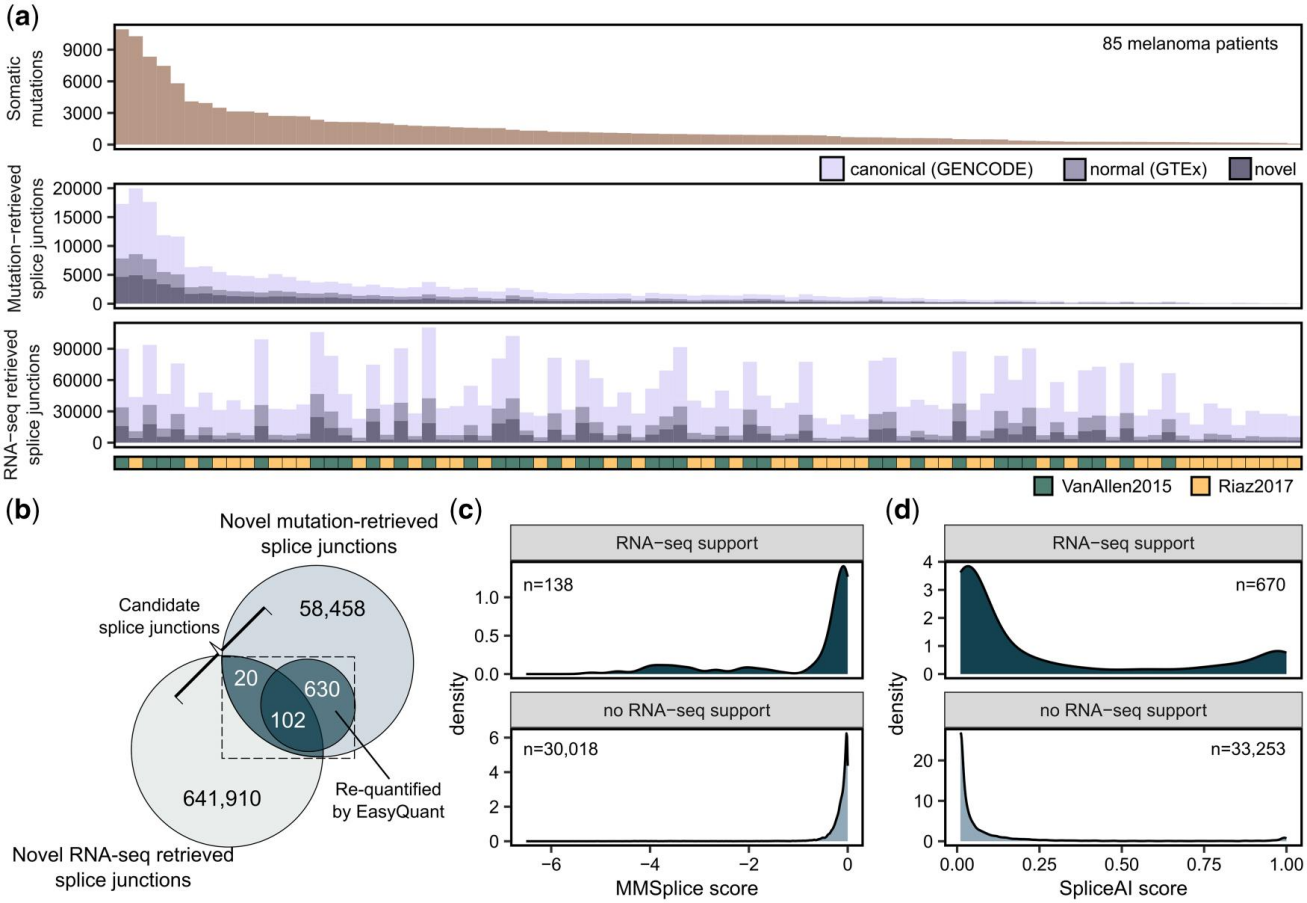
Identification of mutation-retrieved splice junctions supported by RNA-seq in melanoma samples. (a) The number of somatic mutations, mutation-retrieved splice junctions and RNA-seq splice derived junctions per sample in the discovery cohort of 85 melanoma samples. (b) Overlap of mutation-retrieved splice junctions with those that were found in RNA-seq by SplAdder or LeafCutter and those that were requantified in RNA-seq with EasyQuant. Mutation-retrieved splice junctions with RNA-seq support (i.e. by SplAdder, LeafCutter, or EasyQuant) were defined as candidate splice junctions. (c, d) The distribution of (c) MMSplice and (d) SpliceAI scores of candidate splice junctions with RNA-seq support and mutation-retrieved splice junctions without RNA-seq support.

Somatic SNVs and INDELs are truly tumor-specific as they are detected by comparing WES in tumor and matched normal samples. Such mutations can lead to a loss or gain of splicing donor or acceptor sequence motifs. We reasoned that splice junctions can be defined as tumor-specific targets (i) if they are caused by a somatic mutation, (ii) if they are non-canonical and not detected in a large cohort of RNA-seq samples of unmatched healthy tissues (Jaganathan *et al.* 2019), and (iii) if they are detected in the RNA-seq of the patient’s tumor sample. To develop sufficiently stringent detection criteria for such targets, we analyzed matched tumor and normal WES data and tumor RNA-seq data of 85 melanoma samples from two studies (Van Allen *et al.* 2015, Riaz *et al.* 2017) as a discovery dataset using the functionalities of splice2neo and EasyQuant (Figs 1c and 2a).

First, we identified between 93 and 10 931 (median 963) somatic SNVs or INDELs in exonic and intronic regions per sample (Fig. 2a, upper panel). Then, we retrieved the potential effects of the identified somatic mutations on splicing with the deep learning-based tools MMSplice (Cheng *et al.* 2019) and SpliceAI (Jaganathan *et al.* 2019). MMSplice was used to predict if a somatic mutation could cause ES events and SpliceAI was used to predict all potential A3SS, A5SS, ES, and IR events. Both tools report effect scores, which reflect the probability or the effect strength of a mutation to alter splicing. Initially, we did not apply cut-offs on these effect scores, and all potential mutation effects on splicing from MMSplice and SpliceAI, including low scoring effects, were annotated with splice2neo and converted into a common splice junction format (“mutation-retrieved splice junction”) (Fig. 2a, middle panel). Next, we removed canonical splice junctions in reference databases (GENCODE) and normal splice junctions previously detected in RNA-seq of 1740 samples of 53 healthy tissues from the GTEx atlas (The GTEx Consortium 2015, Jaganathan *et al.* 2019). After excluding canonical and normal splice junctions, we considered between 15 and 4952 (median 430) junctions as novel mutation-retrieved splice junctions for further analysis (Fig. 2a, middle panel).

The reported effect scores from MMSplice and SpliceAI were low for most of the novel mutation-retrieved splice junctions (Supplementary Fig. S2A and B) and decreased with increasing distance of the mutation to the junction position (Supplementary Fig. S2C). Although SpliceAI and MMSplice effect scores were correlated for jointly identified ES splice junctions (Pearson correlation *R* = −0.64), there were many splice junctions with divergent splice effect scores (Supplementary Fig. S2D).

Next, we identified splice junctions in matched tumor RNA-seq for the 85 melanoma samples using the RNA-seq-based tools LeafCutter (Li *et al.* 2018) and SplAdder (Kahles *et al.* 2016). The number of detected splice junctions differed strongly between the Van Allen *et al.* (2015) and the Riaz et al. (2017) cohort (Supplementary Fig. S2E and F). Overall, we identified between 16 826 and 110 419 splice junctions per patient, of which between 1597 and 24 480 were novel (i.e. absent from GENCODE and GTEx) (Fig. 2a, lower panel). The percentage of novel splice junctions was higher among mutation-retrieved splice junctions (26%) than among the splice junctions retrieved from RNA-seq (15%) (Supplementary Fig. S2G).

Next, we overlapped the novel mutation-retrieved splice junctions (total *n* = 59 210) with the novel RNA-seq retrieved splice junctions (total *n* = 642 032). For 85 melanoma patients, we identified in total 122 mutation-retrieved splice junctions that were also identified by SplAdder or LeafCutter in RNA-seq (Fig. 2b). We re-evaluated all novel mutation-retrieved splice junctions by quantifying RNA-seq reads supporting the splice junctions or read coverage of retained introns with EasyQuant (Fig. 1b). We observed at least one junction read (A3SS, A5SS, and ES) or a median read coverage > 0 (IR) using this targeted approach for 732 out of 59 210 mutation-retrieved splice junctions (Fig. 2b, Supplementary Fig. S2G and H). Requantified splice junctions and splice junctions identified by SplAdder or LeafCutter had a high overlap of 102 splice junctions (83% of the RNA-seq retrieved splice junctions) (Fig. 2b). Notably, mutation-retrieved junctions supported by RNA-seq were associated with stronger mutation effect scores from SpliceAI and MMSplice (Fig. 2c and d).

For the identification of target splice junctions that are potentially tumor-specific, we focused on the subset of mutation-retrieved splice junctions with RNA-seq support (*n* = 752) for further analysis.

### 3.4 Prediction of target splice junctions in melanoma samples

**Figure 3.**
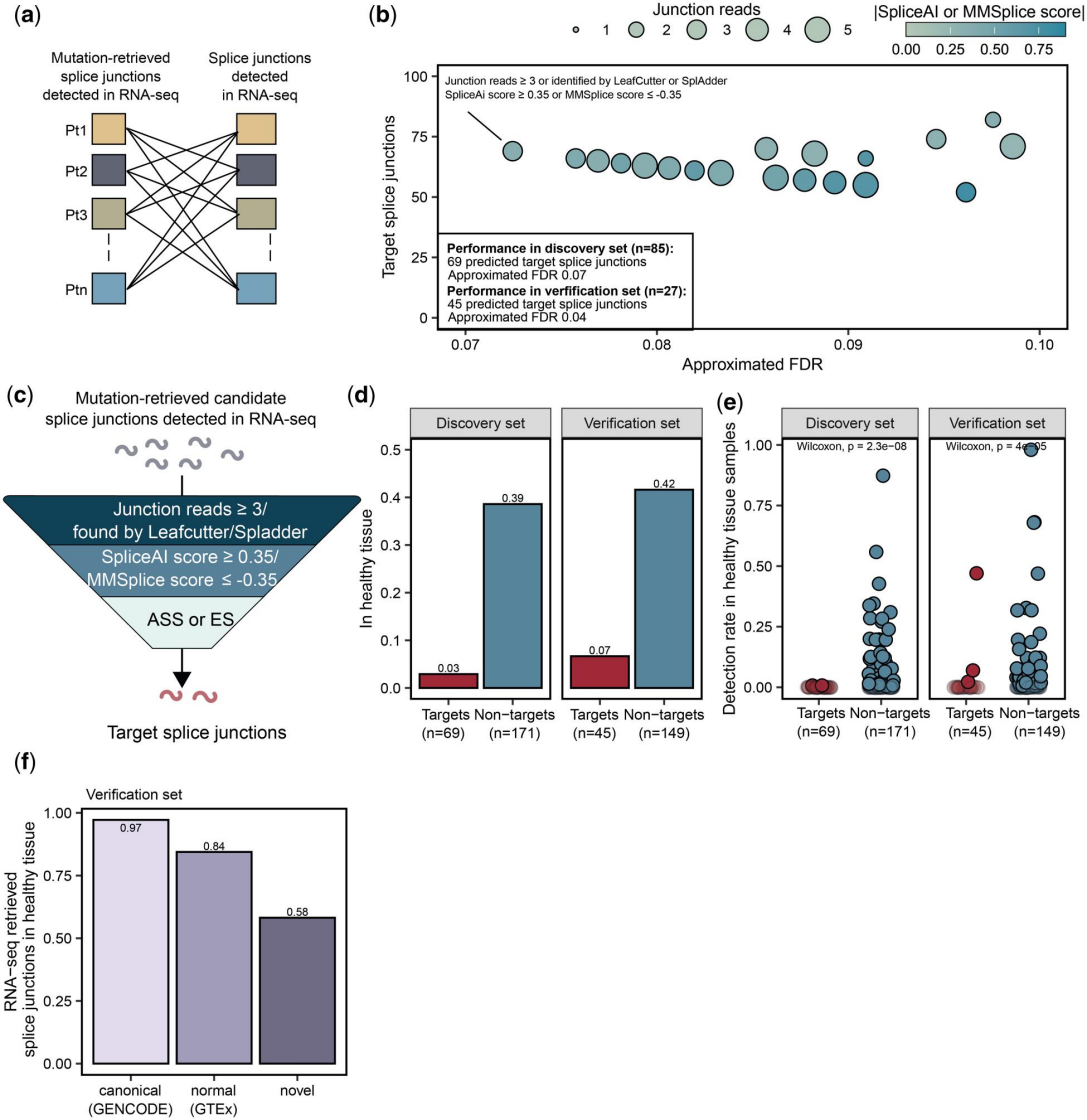
Prediction of tumor-specific target splice junctions based on mutation effect scores and RNA-seq support. (a) To calculate the amount of non-specific junctions and estimate a FDR, candidate junctions were compared to splice junctions identified by SplAdder/LeafCutter in other samples’ RNA-seq and their supporting reads were quantified in RNA-seq data of other samples with EasyQuant. (b) Candidate splice junctions from the discovery set were gradually filtered by thresholds on the mutation effect scores from MMSplice and SpliceAI, and the requantification read support and the resulting estimated FDR and number of target splice junctions was determined. The set of filtering thresholds with lowest estimated FDR was selected as an optimal detection rule for target splice junctions and applied to verification cohort of 27 melanoma samples. This figure shows a subset of data from Supplementary Fig. S4A. (c) Target splice junctions were predicted from candidate junctions by the following detection rule: (i) restriction to ES and ASS events, (ii) SpliceAI score ≥ 0.35 or MMSplice score ≤ −0.35, and (iii) identification by LeafCutter/SplAdder or requantification with at least 3 junction reads by EasyQuant. (d) Fraction of targets and non-targets found in any sample of the healthy tissue cohort. (e) Detection rate of the target and non-target splice junctions with splice junctions identified by SplAdder/LeafCutter in an additional RNA-seq dataset of 141 samples from 49 healthy tissues in the discovery and verification dataset. Transparent data points refer to targets or non-targets not found in any healthy tissue sample. (f) The fraction of splice junctions derived from RNA-seq found in additional RNA-seq dataset of 141 samples from 49 healthy tissues in the discovery and verification dataset.

Next, we wanted to identify stringent detection rules to predict which mutation-retrieved splice junctions with RNA-seq support (“candidates”) are caused by a somatic mutation. To estimate the FDR, we assumed that a junction that is caused by a mutation should not appear in RNA-seq of other samples without the mutation. In a sample permutation analysis, we assessed the support of such candidates in RNA-seq data from all other tumor samples without the corresponding somatic mutation (Fig. 3a, Methods). RNA-seq support in other tumor samples was evaluated by requantification with EasyQuant and with the RNA-seq derived junctions identified in the other samples. Here, we observed that half of the candidate splice junctions from ASS or ES events with RNA-seq support in the actual tumor sample were supported by RNA-seq reads in at least one independent tumor sample, resulting in a high estimated FDR of 0.50. This indicates that many of these candidate splice junctions can occur independently of the somatic mutation and, therefore, might not be tumor-specific and that more stringent filtering is required.

Consequently, we gradually filtered candidate splice junctions by thresholds on the mutation effect scores and the requantification read support (Fig. 3b). First, we focused on splice junctions from ES and ASS events. We estimated the FDR with the sample permutation analysis and selected the set of filtering thresholds with lowest estimated FDR as optimal detection rule for target splice junctions. This optimal detection rule was defined by (i) SpliceAI score ≥ 0.35 or MMSplice score ≤ −0.35 and (ii) identification by LeafCutter or SplAdder or requantification with at least 3 junction reads by EasyQuant (Fig. 3b and c). This detection rule resulted in an estimated FDR of 0.07 and identified 69 target splice junctions for 85 melanoma tumors in the discovery cohort (Supplementary Table S1). We evaluated the detection rule in an independent verification cohort of melanoma patients [Hugo 2016 cohort (Hugo *et al.* 2016)] and predicted 45 splice junctions for 27 patients with an estimated FDR of 0.04 (Supplementary Fig. S3A, B and Table S1).

For IR events no filter combination led to an estimated FDR lower than 0.50 in the discovery and verification cohort (Supplementary Fig. S4), and we, therefore, excluded IR events from further analysis.

To estimate the tumor-specificity of target splice junctions, we compared all candidate splice junctions with splice junctions identified by Leafcutter or SplAdder in an additional independent RNA-seq dataset of 141 samples from 49 healthy tissues (Weber *et al.* 2022). Here, only 2 out of 69 (3%) and 3 out of 45 (7%) splice junctions predicted as targets in the discovery and verification set, respectively, were found in any healthy tissue sample. This was a strong reduction compared to splice junctions that were not predicted as targets (39% in discovery set and 40% in verification set) (Fig. 3d and e). We also used the 141 healthy tissue samples to examine the tumor-specificity of splice junction sets derived from tumor RNA-seq alone. As expected, 97% of the canonical and 84% of the normal RNA-seq derived splice junctions overlapped with the healthy tissue splice junctions. However, also among the novel RNA-seq derived splice junctions, which were already filtered against GTEx, 58% were also found in the independent dataset of healthy tissues (Fig. 3f). Together, this data shows that the here described approach of associating splicing with somatic mutation effects using splice2neo and the detection rule leads to strong enrichment of tumor-specific splice junction targets.

### 3.5 Experimental confirmation of exon skipping junctions in tumor samples

**Figure 4.**
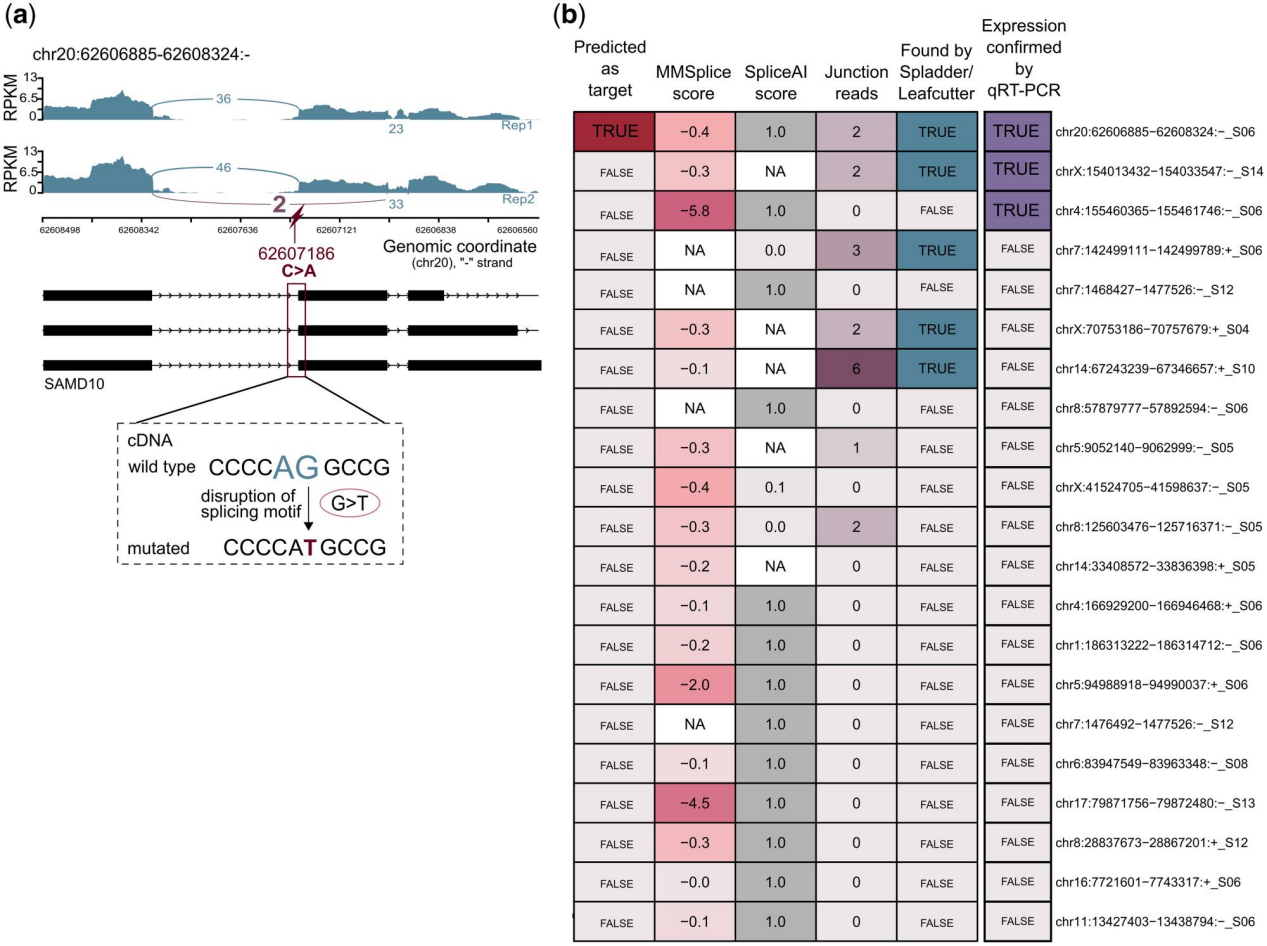
Experimental confirmation of target splice junction from ES. (a) Sashimi plot for the experimentally confirmed splice junction chr20:62606885–62608324:- from an ES event that was predicted as a target. The target splice junctions may be caused by a SNV that changes C to A on the negative strand, resulting in a disruption of an acceptor motif in the cDNA. (b) The RNA expression of 21 splice junctions from ES events were analyzed with qRT-PCR. Heatmap showing MMSplice and SpliceAI score, the number of junction reads, whether a junction was found by an RNA-seq tool (Leafcutter/Spladder) and whether junctions were predicted as targets.

Next, we experimentally confirmed ES targets by qRT-PCR, which were predicted from eight formalin-fixed, paraffin-embedded (FFPE) tumor samples (Weber *et al.* 2022) (Supplementary Fig. S5A, B and Table S2). In total, we tested 21 ES events, of which only one was predicted as a target by the established detection rule (Supplementary Fig. S5B, C, and Table S3). This target splice junction (chr20:62606885–62608324:-) was predicted to be caused by a somatic mutation, disrupting an acceptor motif in the gene *SAMD10* (Sterile Alpha Motif Domain Containing 10) (Fig. 4a). The targeted requantification of RNA-seq reads resulted in only two supporting junction reads, but LeafCutter detected the target splice junction in RNA-seq and the associated mutation resulted in the maximal SpliceAI score of 1.0 (Fig. 4a, Supplementary Table S3), indicating a strong effect on splicing.

The expression of the target splice junction and two additional non-target junctions could be confirmed with qRT-PCR on RNA level (Fig. 4a, Supplementary Table S3).

### 3.6 Splice junctions can generate neoantigen candidates

**Figure 5.**
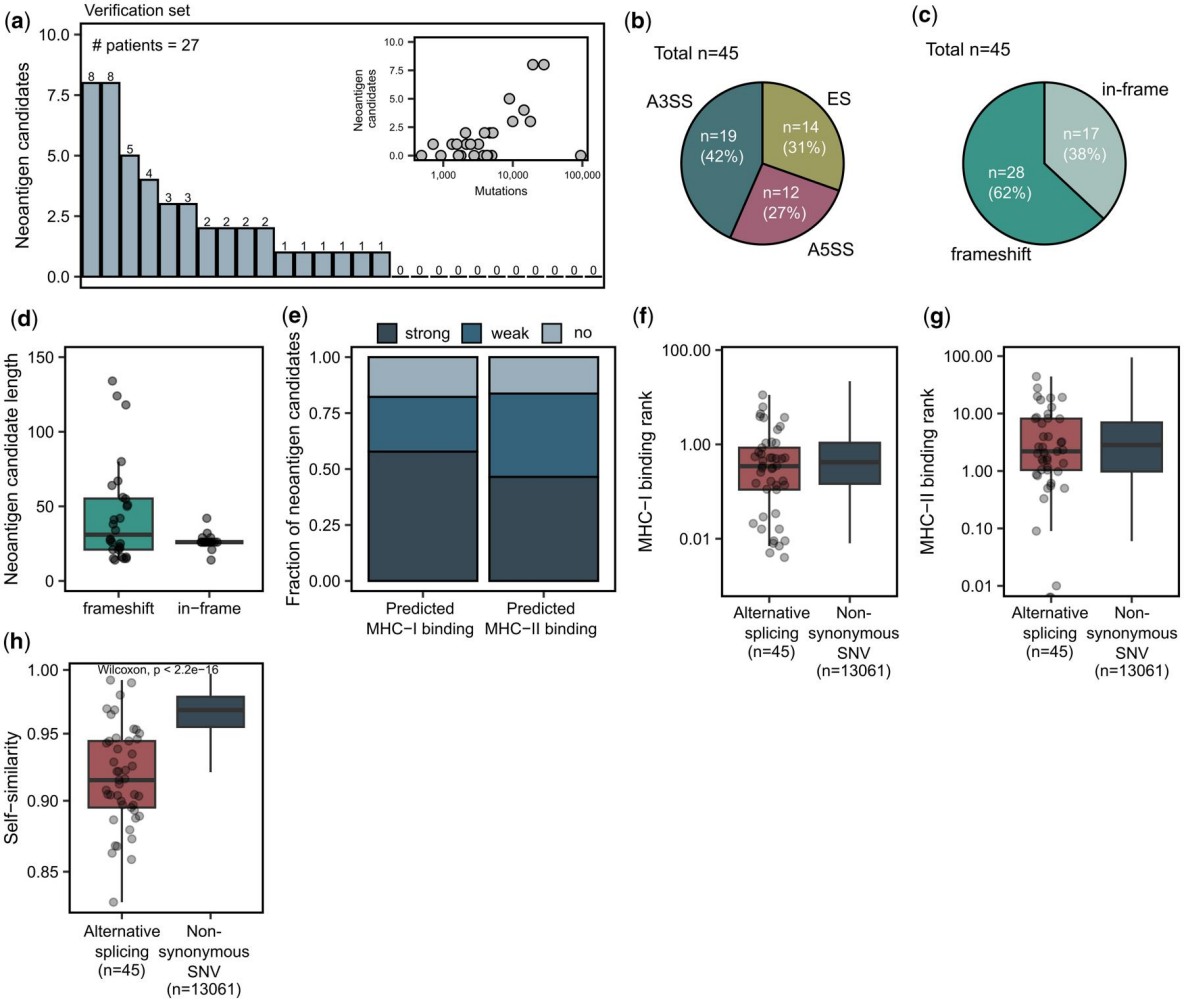
Target splice junctions generate neoantigen candidates. (a) The number of neoantigen candidates per sample in the verification dataset. Inlet: Correlation of the neoantigen candidate count with the tumor mutation burden. (b) The fraction of neoantigen candidates from A3SS, A5SS, and ES events. (c) The fraction of frameshift and in-frame neoantigen candidates. (d) The length distribution of frameshift and in-frame neoantigen candidates. (e) The fraction of strong or weak binding MHC-I and MHC-II neoantigen candidates.(strong: MHC-I binding rank < 0.5, MHC-II binding rank < 2, weak: 0.5 ≤ MHC-I binding rank < 2, 2 ≤ MHC-II binding rank < 10, no: MHC-I binding rank ≥ 2, MHC-II binding rank ≥ 10). (f, g) (f) MHC-I and (g) MHC-II binding rank of the best predicted neoepitope per neoantigen candidate for those from alternative splicing (AS) and SNVs. SNV-derived neoantigen candidates were retrieved as described in (Lang *et al*. 2023). (h) Self-similarity (Bjerregaard *et al.* 2017) of the best predicted neoepitope per neoantigen candidate for those from alternative splicing and those from SNVs.

Next, we examined whether the predicted target splice junctions encode novel peptide sequences and, therefore, qualify as neoantigen candidates. For in-frame junctions, we considered the peptide sequence (±13 amino acids) around the junction and potentially inserted amino acids using splice2neo. For frame-shift junctions, the peptide sequence was extended until the next stop codon. We refer to peptide sequences from predicted tumor-specific targets as “neoantigen candidates.” In total, 42 of the 45 predicted target splice junctions affected CDS and were translated into at least one peptide sequence, resulting in 45 neoantigen candidates for the 27 melanoma patients (Supplementary Table S4). Depending on the mutation load, we identified in average 1.7 neoantigen candidates from alternative splicing per melanoma patient (Fig. 5a). We found that the majority of neoantigen candidates in the verification cohort derived from an A3SS event (42%), followed by ES events (31%) (Fig. 5b). Furthermore, 62% of splicing-derived neoantigen candidates were generated by a frameshift. While also in-frame A3SS or A5SS events could generate neoantigen candidates longer than 26 amino acids if additionally amino acids were inserted, frame-shift splice junctions led generally to longer novel peptide sequences of up to 134 amino acids (Fig. 5c and d).

Next, we predicted MHC-I and MHC-II binding features with NeoFox (Lang *et al.* 2021) using patient-specific MHC-I and MHC-II alleles. We found that 58% of the neoantigen candidates from alternative splicing were predicted to generate at least one strongly binding MHC-I epitope (MHC-I binding rank < 0.5), and 47% of the neoantigen candidates were predicted to generate at least one strongly binding epitope for MHC-II (MHC-II binding rank < 2) (Fig. 5e). Neoantigen candidates from alternative splicing had comparable MHC-I and MHC-II binding ranks to neoantigen candidates derived from non-synonymous SNVs of the same cohort (Fig. 5f and g). However, the best-predicted MHC-I neoepitope per neoantigen candidate from alternative splicing was less self-similar to the wild-type proteome (Bjerregaard *et al.* 2017) compared to the best-predicted neoepitope per neoantigen candidate from nonsynonymous SNVs (Fig. 5h), indicating a stronger potential for immunogenicity in the group of neoantigen candidates derived from alternative splicing.

## 4 Discussion

Disruption of canonical splicing in tumors can generate novel gene products that might be excellent targets for individualized cancer vaccines (Frankiw *et al.* 2019, Smith *et al.* 2019). While RNA-seq can detect thousands of novel splice junctions in individual tumor samples, it is challenging to ensure their specificity to tumor cells (Baralle and Giudice 2017, David *et al.* 2020). One approach is to predict neoantigen candidates from alternative splicing by combining RNA-seq from tumor samples with RNA-seq from matched healthy tissue (Chai *et al.* 2022). Given the diversity of splicing across tissues and cell types, it is questionable which healthy tissue is suitable as a control and if a single normal sample is sufficient. Alternatively, tumor-specificity might be defined by the absence of splice junctions from large healthy tissue sample collections of unrelated subjects (Zhang *et al.* 2020, Pan *et al.* 2023). Databases of canonical reference splice junctions or large RNA-seq sample collections of healthy tissues, such as GTEx (The GTEx Consortium 2015), are valuable resources for constructing exclusion lists. However, it remains unclear if such data is suitable to capture splicing variations in rare cell types or conditions as well as individual splicing events caused, e.g. by rare germline mutations.

In this study, we hypothesized that alternative splicing created by a loss or gain of canonical splicing sequence motifs by somatic mutations are eligible as tumor-specific targets and might be suitable neoantigen candidates for individualized cancer immunotherapy approaches. With splice2neo, we implemented an R-package with several modular functionalities to identify mutation-retrieved splice junctions with support in RNA-seq as candidates for potentially tumor-specific targets for individual cancer patients.

Multiple pipelines were recently described to predict neoantigen candidates from alternative splicing. NeoSplice (Chai *et al.* 2022) and ASNEO (Zhang *et al.* 2020) rely on a single RNA-seq-based method for splicing detection and are end-to-end pipelines from raw files to neoantigen candidates. IRIS (Pan *et al.* 2023) is a modular neoantigen prediction pipeline based on RNA-seq and supports customized pipelines. Regtools (Cotto *et al.* 2023) provides functionalities to integrate DNA sequencing and RNA-seq data to identify potential splice-associated variants. DICAST (Fenn *et al.* 2023) integrates several RNA-seq splicing tools for unified junction analysis. Both, Regtools and DICAST lack the transcript and protein sequence annotation for functional downstream analyses. In contrast to static end-to-end pipelines, splice2neo is designed as a modular library of multiple functionalities for customized splice junction analysis. The functionalities include the unified integration of results from upstream tools, exclusion of canonical or normal junctions, and annotation with transcript and peptide sequences. The implementation of splice2neo as an R-package allows compatibility with multiple other methods from the Bioconductor project (Gentleman *et al.* 2004, Huber *et al.* 2015) for interactive analysis or integration into target identification pipelines. Splice2neo is not limited to the currently supported upstream tools but can be easily extended in the future to support other tools for alternative splicing detection or other event types, such as splicing junctions between exons and transposable elements (Burbage *et al.* 2023, Merlotti *et al.* 2023). The proposed approach works with data from single patients, which is relevant in the context of neoantigen prediction for individualized cancer vaccines but splice2neo might also be used to analyze larger cohorts of tumor samples to identify shared tumor-associated targets.

Besides splice2neo, we developed a novel method called EasyQuant to requantify splice junctions in RNA-seq in a targeted manner. Several specialized tools with a requantification steps exists, such as the STAR in 2-pass mode (Dobin *et al.* 2012), Toblerone (Lonsdale *et al.* 2023) for ES events, or TrinityFusion (Haas *et al.* 2019) and EasyFuse (Weber *et al.* 2022) for gene fusions. In contrast to these tools, requantification by EasyQuant is not restricted to junctions or context sequences retrieved by the respective tool and also not limited to a specific splicing type or mutation type.

Using our novel tools splice2neo and EasyQuant, we initially retrieved novel mutation-retrieved splice junctions that were supported by RNA-seq without any cut-off on splice effect scores. Only if we applied a stringent detection rule on the mutation effect scores and RNA-seq support, we were able to decrease the FDR and enrich for expressed splice junctions that could indeed be associated with somatic mutations. Depending on the somatic mutation burden, on average 1.7 neoantigen candidates from alternative splicing could be predicted per patient with an estimated FDR of 0.04. We assume that the number of neoantigen candidates from somatic mutation-derived splicing is not many magnitudes higher but still can be increased by technical advances. In the future, the computational analysis of larger tumor cohorts from more diverse entities and using personalized reference genomes may allow more sensitive detection rules, e.g. by a machine learning approach.

Studies predicting splicing-derived neoantigen candidates from tumor RNA-seq alone or in combination with matched normal RNA-seq reported markedly higher numbers of non-canonical splice junctions per patient in several tumor entities (Kahles *et al.* 2018, Chai *et al.* 2022, Merlotti *et al.* 2023). However, it remains unclear whether those splice junctions identified from RNA-seq alone are truly tumor-specific and qualify as safe and effective targets in individualized cancer vaccines.

We were not able to show that candidate splice junctions from IR events are potentially tumor-specific. IR detection can be generally challenging as RNA-seq reads might derive from unspliced pre-mRNA, repeat regions, or other transcripts (Middleton *et al.* 2017, Broseus and Ritchie 2020). However, it was shown that somatic mutations indeed can cause IR events (Jung *et al.* 2015), that IRs can generate MHC-I binding epitopes (Smart *et al.* 2018) and that the load of non-canonical peptides derived from IRs correlated with favorable prognosis in pancreatic cancer (Dong *et al.* 2022). These observations suggest that also IRs could contribute to the neoantigen repertoire. Improved computational tools for IR detection and long read sequencing might allow to better investigate splice junctions from IRs in the future (Middleton *et al.* 2017, Broseus and Ritchie 2020, Lorenzi *et al.* 2021, Oka *et al.* 2021).

Here, we showed that the predicted target splice junctions from somatic mutations can generate neoantigen candidates. By association with somatic mutations, the number of these neoantigen candidates depends on the tumor mutation burden. Therefore, neoantigen candidates from mutation-derived splicing might be in particularly rare in tumor entities with low tumor mutation burden which still could be sufficient for anti-tumor response as long as they are of high quality as shown in pancreatic cancer (Balachandran *et al.* 2017, Levink *et al.* 2021). Indeed, those targets frequently cause frameshifts and lead to longer novel peptide sequences, leading to strong MHC binding neoepitope candidates that are dissimilar to the wild-type proteome. These features may characterize splicing-derived neoantigen candidates as promising targets for individualized cancer vaccines (Lang *et al.* 2022). Future studies are required to examine if the identified neoantigen candidates indeed mount functional T-cell responses upon individualized cancer vaccines.

## Supplementary Material

vbae080_Supplementary_Data

## Data Availability

RNA-seq data for the FFPE cohort (Weber *et al.* 2022) is available in European Genome-phenome Archive (EGA) under accession number EGAS00001004877 and WES was uploaded as EGAS00001007589. Data for the Hugo cohort (Hugo *et al.* 2016) is available in the Sequence Read Archive (SRA) under accession numbers SRP067938, SRP090294 (WES-seq) and SRP070710 (RNA-seq). Data for the Riaz cohort (Riaz *et al.* 2017) is available under accession numbers SRP095809 (WES-seq) and SRP094781 (RNA-seq). Data for the Van Allen cohort (Van Allen *et al.* 2015) is available in dbGap under accession number phs000452.v2.p1. RNA-seq for the cohort of healthy tissue is available under is in SRA under NCBI BioProject ID PRJNA764684. The source code and documentation of the tools splice2neo (https://github.com/TRON-Bioinformatics/splice2neo) and EasyQuant (https://github.com/TRON-Bioinformatics/easyquant) are available under open source licenses on GitHub. The scripts for the analysis of the sequencing data are provided in a separate repository (https://github.com/TRON-Bioinformatics/splicing_manuscript_scripts).
