# Supplementary material for: Prediction of tumor-specific splicing from somatic mutations as a source of neoantigen candidates
Source: Bioinform Adv. 2024 May 29;4(1):vbae080. doi: 10.1093/bioadv/vbae080 (PMC11165244; doi:10.1093/bioadv/vbae080)
Supplement: vbae080_Supplementary_Data [file vbae080_supplementary_data.zip › Additional_file_1_v10.pdf]

Supplementary Methods

Whole exome sequencing

Genomic DNA was isolated from 10µm curls of FFPE tissue using the QIAamp DNA FFPE Tissue Kit (Qiagen) or Maxwell RSC DNA FFPE Kit (Promega) according to the manufacture's protocol. The DNA quantity was assessed using Qubit 3 fluorometer using the dsDNA HS Assay Kit (Invitrogen).

Whole exome libraries were prepared in duplicates with an input of 100 ng genomic DNA from FFPE tissue samples. The genomic DNA was fragmented to a size range of 200 ± 50 bp using the Covaris S220 instrument. The fragmented DNA was end-repaired and adenylated followed by ligation of sequencing adaptor and the appropriate eight-nucleotide NEXTFLEX DNA barcode (Perkin Elmer) and pre-amplification of the library using the KAPA Hyper Prep kit (Roche). Subsequently, target regions were hybridized to biotinylated baits (SureSelectXT Human All Exon v6 and SureSelect XT Reagent kit, Agilent) and isolated using streptavidin-coated magnetic beads (Invitrogen). The post-capture library was amplified with the KAPA Library Amplification kit (Roche). Final library quantity and quality was assessed using the Qubit 3 fluorometer with Qubit dsDNA HS Assay Kit (Invitrogen) and the BioAnalyzer with High Sensitivity DNA Kit (Agilent).
All libraries were sequenced in paired-end mode (2 x 50 nt) on an Illumina NovaSeq 6000 instrument resulting in around 150 million distinct sequencing reads per library.

Somatic mutation calling

For WES read alignment and somatic mutation calling, we used the tronflow pipelines [55–57] implemented in Nextflow [58]. In short, reads from WES tumor and normal sequencing were aligned to the reference genome hg19 using bwa [59] (v0.7.17). Base score recalibration was performed with GATK4 [60] (v4.2.0.0) and duplicated reads were removed with Picard (GATK4 v.2.0.0). Single nucleotide variants (SNVs) and short insertions and deletions (INDELs) were detected with Mutect2 [61] (GATK4 v.2.0.0) without restriction to any target region and using the GnomAD [62] as a resource of germline mutations. Only mutation calls with PASS in the filter column were considered and calls from individual replicates were combined per sample.

Confirmation of splice junction expression with qRT-PCR

Primers were designed as described previously [7]. In short, context sequences as predicted by splice2neo were used for primer design with Primer-BLAST [68]. Primer design was guided by the following restrictions: (1) no primer should align within 20 bp upstream and downstream

of the junction; (2) amplicons must span the junction; and (3) amplicon size should not exceed 150 bp. Primer pairs aligning to off-target loci were removed.

Splice junctions were validated in previously extracted total RNA from fresh frozen tissue samples [7]. The RNA was reverse transcribed into cDNA using TAKARA PrimerScript RT Reagent Kit with gDNA Eraser. Reverse Transcriptase was replaced with water in no amplification controls.

qRT-PCR was performed on a BioRad CFX384 instrument using BioRad SsoAdvanced Universal SYBR Green Supermix. After polymerase activation for 30 s at 98°C, qPCR were run in 40 cycles of two-step PCR with denaturation for 10 s at 98°C and annealing/elongation for 30 s at 60°C.

The PCR products were analyzed for the presence of amplicons matching the expected sizes using QIAGEN's QIAxcel Advanced capillary gel electrophoresis instrument with a 15 bp/600 bp alignment marker.

A custom R script was used to evaluate qRT-PCR and capillary gel electrophoresis results and to assign each tested amplicon as positive or negative given a defined set of rules. (1) The amplicon had to be detected in qRT-PCR with a Ct value below or equal to 35 and at least 5 cycles earlier compared to water and no amplification controls. (2) The amplicon product size had to match the expected amplicon size (allowing a maximum difference of 15% in amplicon size). (3) The amplicon associated peak had to cover at minimum 15% height relative to the total peak heights. Amplicons fulfilling the first two rules but with a relative height below 15% were classified manually by an expert user.

Read support visualization with sashimi plots

A Sashimi plot (Additional File: Fig. S5D) was generated with the python package rmats2sashimiplot (v 2.0.4) (https://github.com/Xinglab/rmats2sashimiplot) using RNA-seq BAM files with coordinate and GENCODE (v34lift37) annotation. The original Sashimi plot was manually modified to include one further affected transcript while a non-affected transcript was removed from the plot.

Supplementary Figures

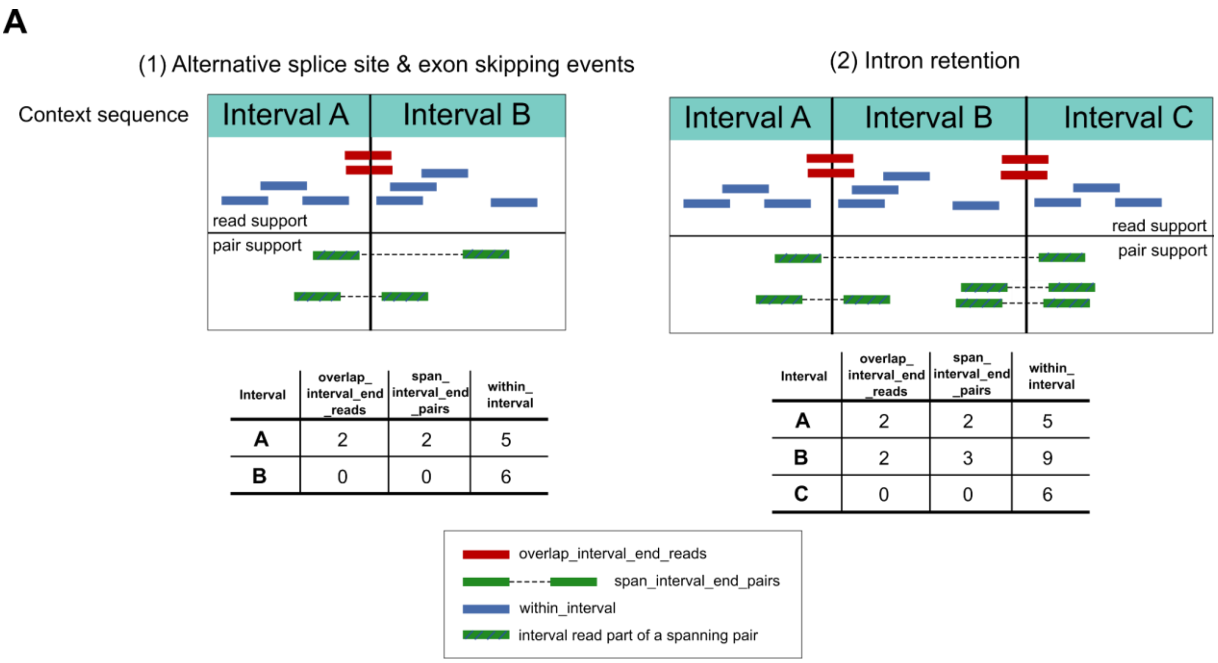

Fig. S1: Re-quantification with EasyQuant in the interval mode.) EasyQuant divides a given context sequence into intervals based on provided coordinates. Both context sequence and interval coordinates can be determined with splice2neo. Upon read alignment to the context sequence, EasyQuant counts reads that overlap the interval ends (“overlap_interval_end_reads”, “span_interval_end_pairs”) or that map into an interval (“within_interval”) and calculates the median and mean coverage of the intervals. In case of junctions derived from alternative splice sites or exon skipping events the context sequence is divided into two intervals and overlap_interval_end reads represent the number of reads covering the junction of interest (“junction reads”). In case of intron retention, the interval is divided into three intervals. Here, the read coverage of the interval located in the middle represents the coverage of the intron of interest.

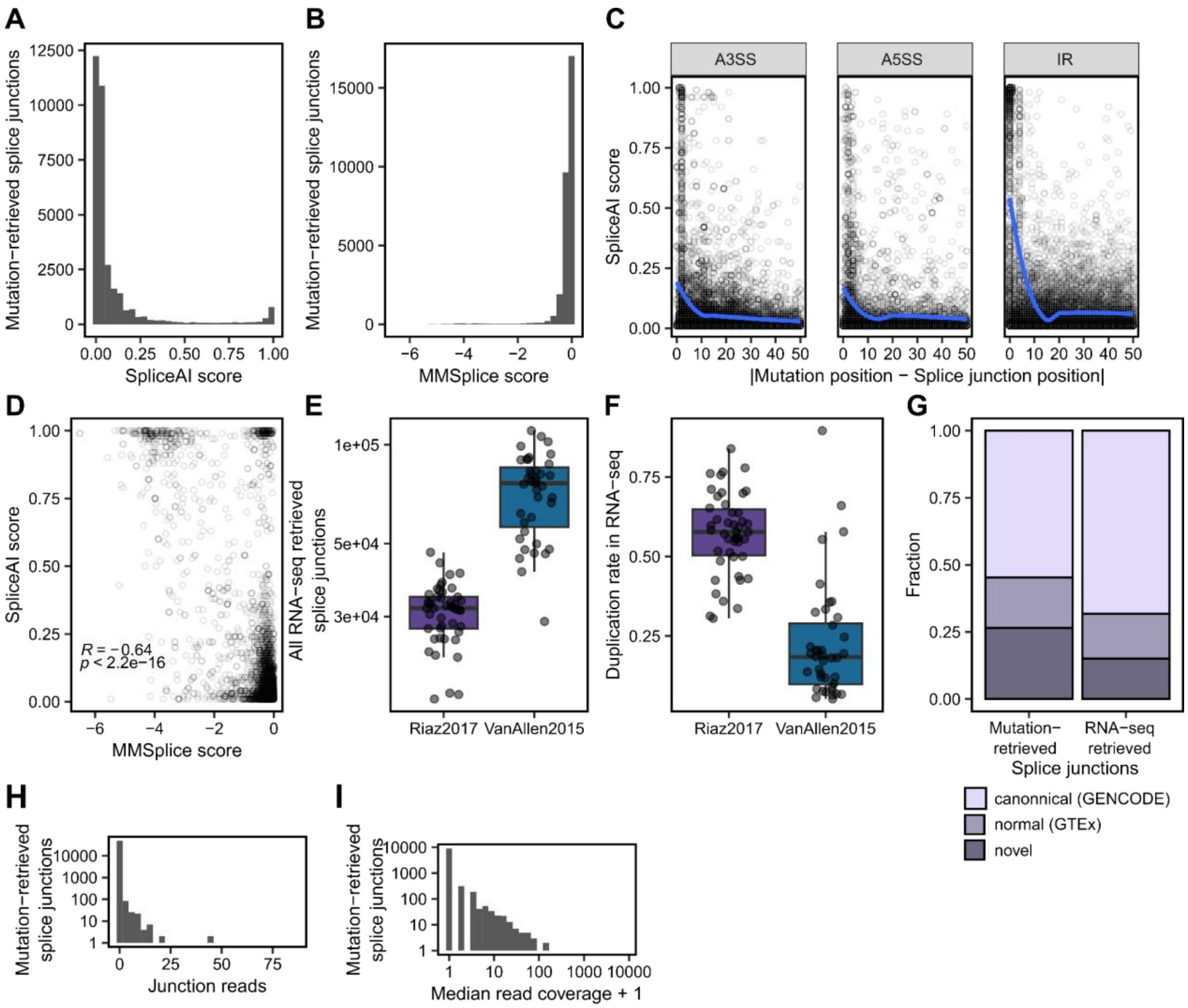

Fig. S2: Overview of the discovery set. (A (A-B) The distribution of **(A)** SpliceAI and **(B)** MMSplice scores in the discovery set. **(C)** The distribution of SpliceAI score dependent on the genomic distance between splice junctions from A3SS, A5SS and IR events and the mutation they were retrieved from. **(D)** Correlation of MMSplice and SpliceAI score for splice junctions from exon skipping events. Negative MMSplice scores indicate stronger exon exclusion effects. **(E)** The number of non-canonical junctions derived from RNA-seq per sample in the Riaz2017 and VanAllen2015 cohorts. **(F)** The duplication rate in RNA-seq per sample in the Riaz2017 and VanAllen2015 cohorts. **(G)** The fraction of splice junctions found in GENCODE and GTEx, found in GENCODE or GTEx and novel junctions among splice junctions derived from RNA-seq and mutation-derived splice junctions. **(H-I)** The distribution of **(H)** number of junction reads of junctions from A3SS, A5SS or ES events and **(I)** the median coverage of predicted retained introns.

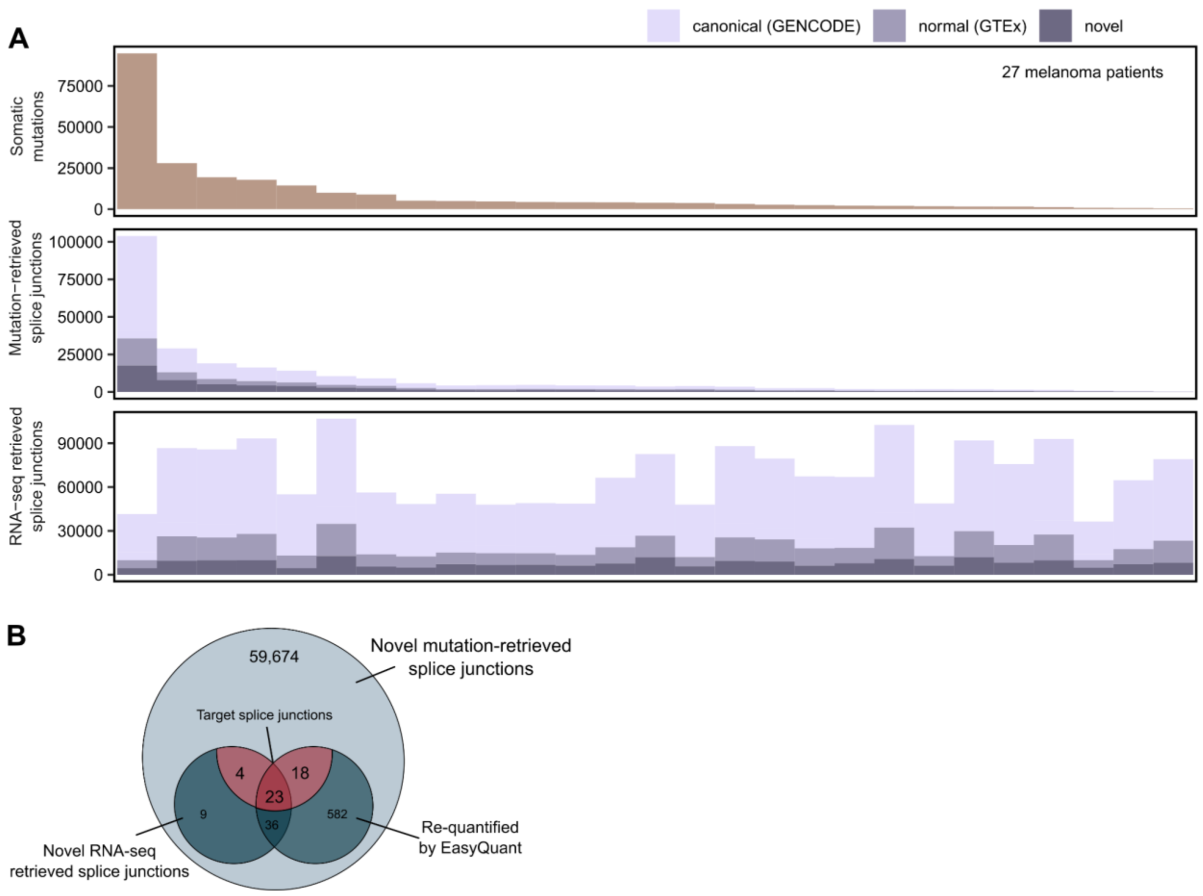

Fig. S3: Overview of the verification set. (A) The number of somatic mutations, mutation-derived splice junctions and RNA-seq derived splice junctions per sample in the verification cohort of 27 melanoma samples. **(B)** Overlap of mutation-retrieved splice junctions with those that were retrieved from RNA-seq by Spladder or Leafcutter, those that were re-quantified in RNA-seq with Easyquant and those that were predicted as targets.

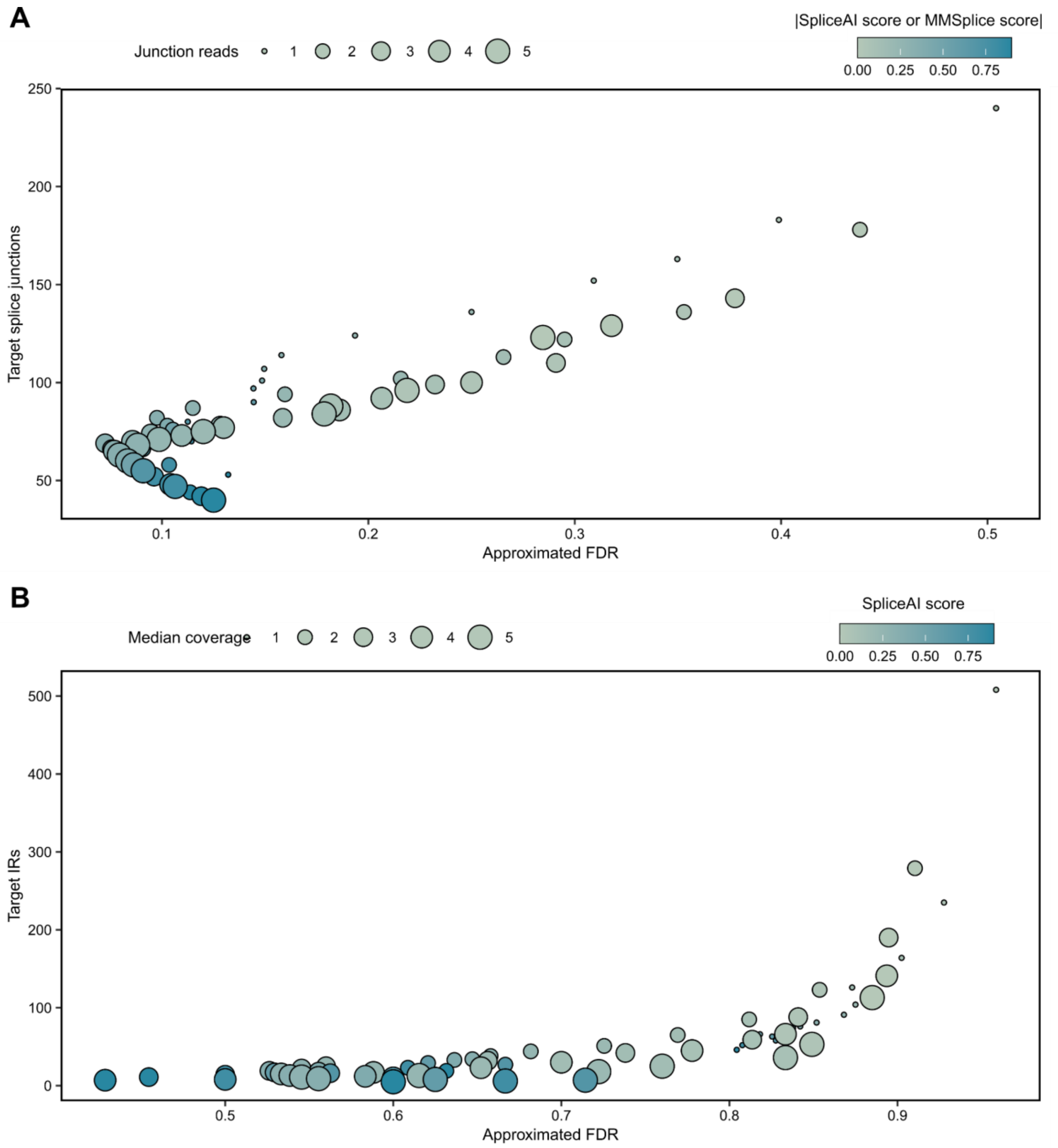

Fig. S4: Estimation of false discovery rate (FDR) and number of targets. (A) Candidate splice junctions by alternative splice site or exon skipping from the discovery set were gradually filtered by thresholds on the mutation effect scores from MMSplice and SpliceAI, and the re-quantification read support and the resulting estimated FDR and number of target splice junctions was determined. Fig. 3B is a subset of this plot. **(B)** Candidate IR events from the discovery were gradually filtered by thresholds on the mutation effect scores from and SpliceAI, and the re-quantification read support and the resulting approximated FDR and number of IR targets was determined.

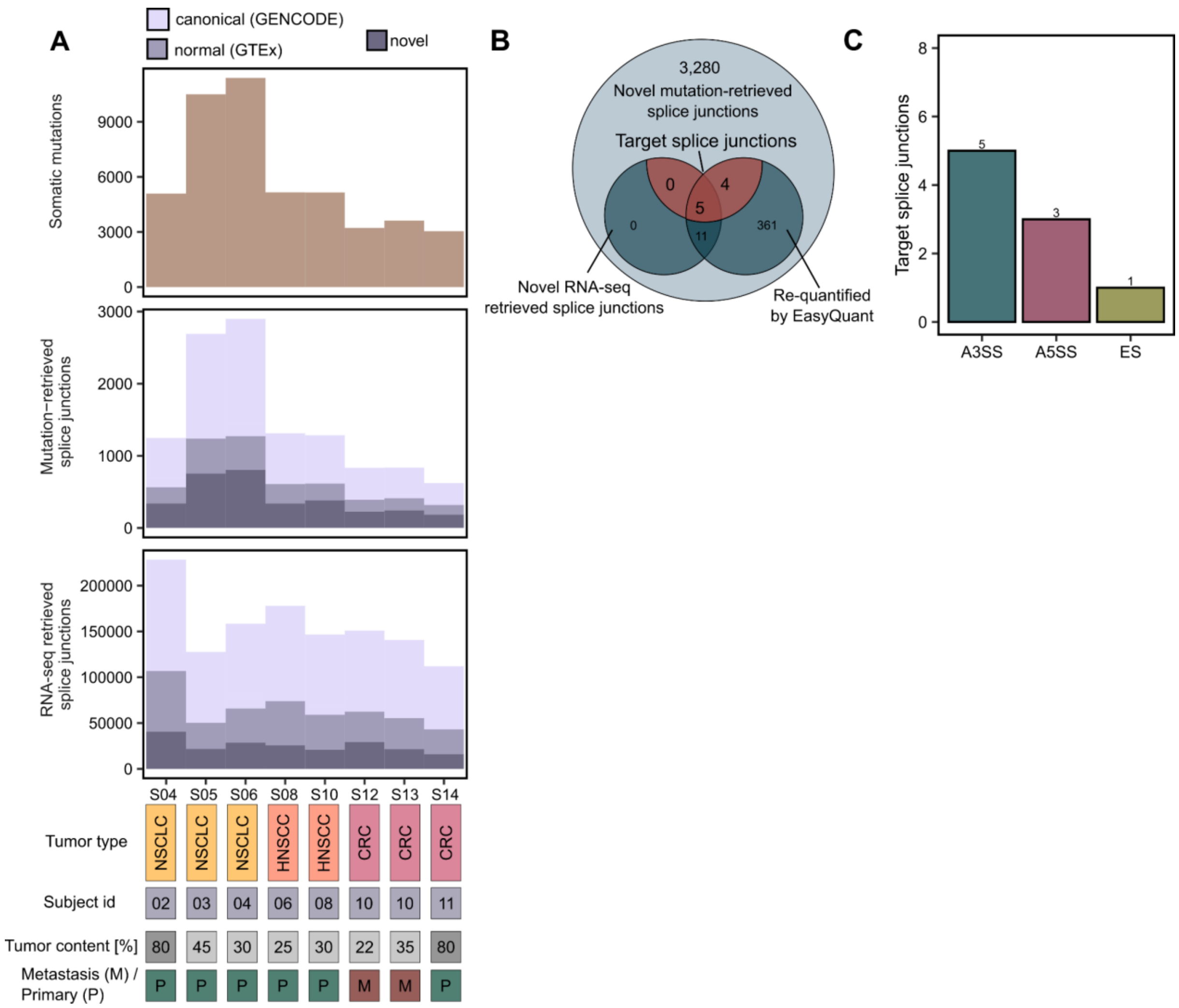

Fig. S5: Overview of the FFPE cohort. (A) The number of somatic mutations, mutation-derived splice junctions and RNA-seq derived splice junctions per sample in the FFPE cohort. **(B)** Overlap of mutation-retrieved splice junctions with those that were retrieved from RNA-seq by Spladder or Leafcutter, those that were re-quantified in RNA-seq with Easyquant and those that were predicted as targets. **(C)** The number of target splice junctions from A3SS, A5SS and ES events.
